# Periodically Pulsed Immunotherapy in a Mathematical Model of Tumor, CD4^+^ T Cells, and Antitumor Cytokine Interactions

**DOI:** 10.1155/2017/2906282

**Published:** 2017-11-09

**Authors:** Hsiu-Chuan Wei, Jui-Ling Yu, Chia-Yu Hsu

**Affiliations:** ^1^Department of Applied Mathematics, Feng Chia University, Seatwen, Taichung 40724, Taiwan; ^2^Department of Financial and Computational Mathematics, Providence University, Shalu Dist., Taichung 43301, Taiwan

## Abstract

Immunotherapy is one of the most recent approaches for controlling and curing malignant tumors. In this paper, we consider a mathematical model of periodically pulsed immunotherapy using CD4^+^ T cells and an antitumor cytokine. Mathematical analyses are performed to determine the threshold of a successful treatment. The interindividual variability is explored by one-, two-, and three-parameter bifurcation diagrams for a nontreatment case. Numerical simulation conducted in this paper shows that (i) the tumor can be regulated by administering CD4^+^ T cells alone in a patient with a strong immune system or who has been diagnosed at an early stage, (ii) immunotherapy with a large amount of an antitumor cytokine can boost the immune system to remit or even to suppress tumor cells completely, and (iii) through polytherapy the tumor can be kept at a smaller size with reduced dosages.

## 1. Introduction

Cancer is one of the most common life-threatening diseases in the 21st century. For example, melanoma is estimated to cause the death of 10,130 people annually in the United States. In 2016, an estimated 76,380 melanoma cases were identified to be invasive; of the 76,380 cases, approximately 46,870 were men and 29,510 were women [1]. Cancer cells can not only divide and grow abnormally at the primary site but also invade other parts or organs of the body through blood circulation or the lymphatic system.

Standard therapies for melanoma are surgical resection, radiation therapy, and chemotherapy. Surgery may remove the tumor, but it often needs subsequent surgery to reduce the risk of recurrence. To reduce the rate of tumor recurrence, wide excision is required at the site of the original lesion; this usually leaves a noticeable scar and may result in treatment failure. Moreover, surgery may be difficult for large lesions or in some areas of the body, such as the face and eyelids [2].

Radiation therapy is often used after surgical resection for patients with locally advanced melanoma or those with inoperable distant metastases. Although this therapy can reduce the rate of local recurrence, it does not compromise survival [3]. Chemotherapy involves the use of drugs to kill cancer cells by stopping or slowing the fast growth of cancer cells. However, as a side effect, chemotherapy also harms healthy cells that grow and divide rapidly under normal circumstances, such as blood cells and cells in the digestive tract. Chemotherapy-related toxicities can occur acutely, subacutely, chronically, or many years after the treatment [4]. To develop treatments with low toxicity, immunotherapies utilizing a patient's own immune system were created [5, 6].

Cancer immunotherapies have considerably focused on the antitumor activities of white blood cells, especially T cells, natural killer (NK) cells, and macrophages. Among them, adoptive cellular therapy (ACT) that uses CD8^+^ T cells is considered a powerful treatment against established tumors. However, a few studies have addressed tumor-specific CD4^+^ T cells. CD4^+^ T cells have traditionally been regarded as helpers to achieve full activation of tumor-specific cytotoxic T lymphocytes (CTLs) [7, 8].

Most cancer immunotherapies have focused on CD8^+^ T cells that recognize a specific antigen and bind to the complex of class I major histocompatibility (MHC) proteins. However, recent evidence has shown that many tumors are immunoselected to evade recognition by CTLs [9]. Therefore, the use of CD4^+^ T cells to fight cancer has been proposed as an alternative approach and proven to have promising effects.

Various studies have demonstrated the ability of CD4^+^ T cells to reject CTL-resistant, MHC class II-negative tumors [9, 10]. Unlike CD8^+^ T cells, which mediate the clearance of pathogens by destroying all infected host cells, CD4^+^ T cells secrete antiangiogenic cytokines to activate other T cells and recruit other tumoricidal myeloid cells to the tumor site [9–11]. Because these destructive mechanisms of CD4^+^ T cells are not affected by the expression level of MHC proteins, they have more advantages in tumor killing than the established effector functions of CD8^+^ T cells [10]. Increasing evidence has demonstrated that the adoptive transfer of tumor-specific CD4^+^ T cells can exert a stronger antitumor effect than CD8^+^ T cells in cancer therapy [4, 12].

There are mathematical theoretical studies about tumor-immune dynamics. Eftimie et al. [13] presented a comprehensive overview on the research in this area. In 2010, they proposed models of a tumor, CD4^+^ T cells, and cytokine interactions to discuss the role of CD4^+^ T cells in melanoma rejection, where CD4^+^ T cells were separated into Th1 and Th2 cells [14]. [15] Anderson et al. explored the dynamics of tumors, CD4^+^ T cells, and antitumor interactions with or without continuous infusion of CD4^+^ T cells or antitumor cytokines produced by Th2. CD4^+^ T cells were grouped as one type of cells, and only the effect of tumor suppressor cytokines, such as interleukin IL-4, was considered. The interaction between CD4^+^ T cells and tumor cells is indirect in the sense that the tumor is suppressed through the action of cytokines. Other theoretical investigations about the tumor-immune dynamics have been mentioned in [9, 10, 16–18].

Anderson et al. [15] discussed the effect of the continuous infusion of CD4^+^ T cells or cytokines produced by Th2 on tumor rejection. In this study, we explore the capacity of periodically pulsed therapies to fight cancer. We turned the continuous system of ordinary differential equations into a discrete dynamical system for the bifurcation analysis of periodically pulsed therapies. Instead of using the typical numerical method for the bifurcation analysis [19], we adopted the adaptive grid technique. Different from the conventional numerical method for the bifurcation analysis, this method is easy to implement because it does not use the continuation technique, normal form, or an initial point to start the trace of bifurcation curves [20, 21].

In Sections 2–5, we will first review the mathematical model by Anderson et al. [15]. The numerical study for a nontreatment case is shown in Section 3. In Section 4, we explore the effect of periodically pulsed therapies delivered at the tumor site. The stability condition of each tumor-free fixed point is analyzed and their dynamics and biological implications are presented.  Section 5 provides a brief summary and discussion.

## 2. The Mathematical Model

Although tumor antigen-stimulated CD4^+^ T cells can produce IFN-*γ* (Th1) or IL-4 (Th2) and both these cytokines can recruit other tumoricidal cells to the tumor site [8, 9, 22], tumor rejection is mediated by IL-4 [10]. Some investigations have reported that Th2 cells are more advantageous compared with Th1 cells in the eradication of CTL-resistant tumors [9, 10]. A human clinical trial on a vaccine also showed that overall recovery from cancer is related to the ratio of vaccine-induced Th2 immunity and antitumor Th2 cells can be found in cancer patients [10, 23, 24]. Based on these data showing the potential importance of CD4^+^ T cells combined with Th2-related antitumor cytokine interactions in tumor clearance, we investigated how these diverse mechanisms interact with periodically pulsed therapies to dictate the antitumor function in this setting.

Let *x*, *y*, and *z* denote tumor cells, CD4^+^ T cells, and antitumor cytokine (IL-4 or any antitumor cytokine produced by Th2), respectively. The proposed model is given as follows:

(1) dxdt=rx1−xK−δxzm+x,dydt=βxyk+x−ay+l1t,dzdt=αxyb+x−μz+l2t,

where *r*(1 − *x*/*K*) is the per capita tumor growth rate and *δxz*/(*m* + *x*) denotes the loss of tumor cells caused by the antitumor cytokine. The fraction *βxy*/(*k* + *x*) is the proliferation of CD4^+^ T cells through interactions with tumor cells, and *αxy*/(*b* + *x*) denotes the production of antitumor cytokines secreted by CD4^+^ T cells. The expression *ay* is the apoptosis (natural death) of T cells, *μz* denotes the loss of cytokine, and *l*_1_(*t*) and *l*_2_(*t*) are immunotherapy treatments that may be time dependent.

A logistic growth equation for tumor cells and Michaelis-Menten kinetics for all the functional forms with different half saturation constants are used. Consequently, it is assumed that the tumor's growth is limited and the production of CD4^+^ T cells and the antitumor cytokine due to tumor cells is also limited.

All the parameters, *r*, *K*, *m*, *δ*, *β*, *k*, *a*, *α*, *b*, and *μ*, are positive constants. *l*_1_⩾0 and *l*_2_⩾0 denote treatments of CD4^+^ T cells and the antitumor cytokine per unit time, respectively. The parameters and their biological interpretations are summarized in Table 1. The time unit is one day and all populations have the unit of volume.

We will first explore the dynamics of the model (1) by one-, two-, and three-parameter bifurcation diagrams for nontreatment.

## 3. Numerical Study for No Treatment Case

According to the analytical study by Anderson et al. [15], there are three equilibriums for a nontreatment case: *E*_0_(0,0, 0), *E*_1_(*K*, 0,0), and the interior steady state *E*^*∗*^(*x*^*∗*^, *y*^*∗*^, *z*^*∗*^), where *x*^*∗*^, *y*^*∗*^, *z*^*∗*^ > 0. The steady states *E*_0_(0,0, 0) and *E*_1_(*K*, 0,0) exist for all parameter values. The equilibrium *E*_0_, where the population is zero, is always a local saddle point. The equilibrium *E*_1_ is asymptotically stable if the antigenicity of the tumor (*β*) is less than a critical value (*β*_*c*_) and a saddle point if *β* > *β*_*c*_, where

(2) $$\begin{array}{c} \beta_{c}=\frac{a\left(k+K\right)}{K}. \end{array}$$

The unique interior equilibrium *E*^*∗*^(*x*^*∗*^, *y*^*∗*^, *z*^*∗*^) exists if and only if *β* > *β*_*c*_, where *x*^*∗*^ < *K*. It means that the tumor size of the interior steady state is always smaller than its carrying capacity [15].

To study the stability of *E*^*∗*^ numerically, we use *l*_2_(*t*) = 0 in the third equation of (1), which is equivalent to

(3) $$\begin{array}{c} \frac{1}{\mu }\frac{dz}{dt}=\frac{1}{\mu }\frac{\alpha xy}{b+x}-z. \end{array}$$

**Figure 1:**
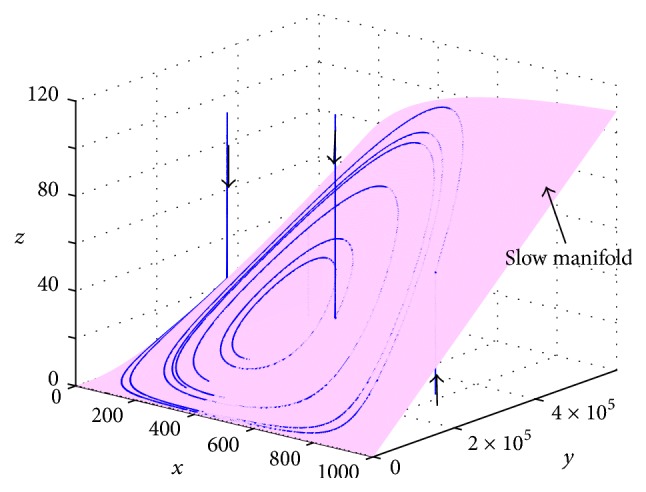
The slow manifold *z* = (1/*μ*)*αxy*/(*b* + *x*) with some selected trajectories.

When the value of *μ* is large, *dz*/*dt* is large unless (1/*μ*)*αxy*/(*b* + *x*) − *z* ≈ 0. Any trajectory tends to approach the surface *S*(*x*, *y*) = (1/*μ*)*αxy*/(*b* + *x*) rapidly and remains close to the surface, where *S*(*x*, *y*) refers to a slow manifold.  Figure 1 shows this phenomenon.

Because the positive equilibrium *E*^*∗*^ is located on the slow manifold, *dz*/*dt* > 0 if *z* < *S*(*x*, *y*) and *dz*/*dt* < 0 if *z* > *S*(*x*, *y*). The stability property of *E*^*∗*^ can be obtained from the following reduced system:

(4) dxdt=rx1−xK−δxSx,ym+x,dydt=βxyk+x−ay.

Let $\bar{E}{}^{*}$ be the positive equilibrium of (4). Then,

(5) $$\begin{array}{c} {\bar{E}}^{*}=\left(\;\frac{ka}{\beta -a},\frac{r\mu }{K\alpha \delta x}\left(\;m+x\;\right)\left(\;b+x\;\right)\left(\;K-x\;\right)\;\right). \end{array}$$

The Jacobian matrix at ${\bar{E}}^{*}$ is

(6) JE¯∗=x−rK−m+xδ∂S/∂x−δSm+x2−δxm+x∂S∂ykβyk+x20.

The determinant of $J({\bar{E}}^{*})$ denoted by $\det\left(J({\bar{E}}^{*})\right)$ is positive because ∂*S*/∂*y* = (1/*μ*)*αx*/(*b* + *x*) > 0. The trace of $J({\bar{E}}^{*})$ denoted by $trace(J({\bar{E}}^{*}))$ can be simplified as

(7) $$\begin{array}{c} -\frac{rx}{K}\left(\;1+\frac{b\left(K-x\right)}{x\left(b+x\right)}-\frac{K-x}{m+x}\;\right). \end{array}$$

When *m* is large, we have $trace(J({\bar{E}}^{*}))<0$. For example, (*K* − *x*)/(*m* + *x*) < 1 if *m* > *K* and thus $trace(J({\bar{E}}^{*}))<0$. So, ${\bar{E}}^{*}$ is stable if *m* is large. A Hopf bifurcation occurs if $trace(J({\bar{E}}^{*}))=0$ because $\det\left(J({\bar{E}}^{*})\right)>0$. Therefore, the parameter values satisfy

(8) $$\begin{array}{c} 1+\frac{b\left(K-x\right)}{x\left(b+x\right)}-\frac{K-x}{m+x}=0 \end{array}$$

at a Hopf bifurcation point, where *x* = *ka*/(*β* − *a*). The stability condition is summarized in the following theorem.

**Theorem 1 .**

The positive equilibrium ${\bar{E}}^{*}$ is locally stable if *m* is large or if the parameter values satisfy

(9) $$\begin{array}{c} 1+\frac{b\left(K-x\right)}{x\left(b+x\right)}-\frac{K-x}{m+x}>0, \end{array}$$

where *x* = *ka*/(*β* − *a*) > 0.

Numerical examples showing the dynamics of small or moderate value of *m* will be presented by using the one- and two-parameter bifurcation diagrams, which will be addressed at the end of this section.

**Figure 2:**
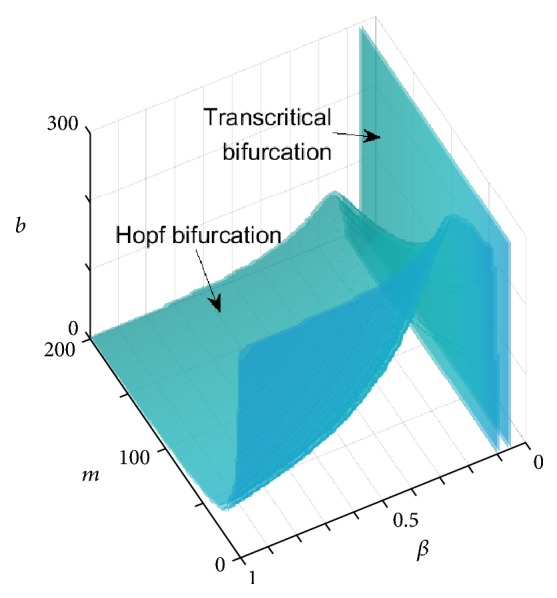
A three-parameter bifurcation diagram using *β*, *m*, and *b* as bifurcation parameters. Parameter values are taken from [15], where *α* = 0.01, *a* = 0.03, *b* = 10^2^, *δ* = 0.1, *k* = 10^3^, *μ* = 50, *m* = 10^2^, *K* = 10^3^, *r* = 0.01.

A natural question that follows from the aforementioned study is how other key parameters, such as the maximum CD4^+^ T cell production rate (antigenicity of the tumor) (*β*), half saturation constant of the tumor killing rate (*m*), and half saturation constant of the antitumor cytokine production rate (*b*), affect the system. To observe these effects, we let *β*, *m*, and *b* be the bifurcation parameters and the parameter domain be [*m*, *β*, *b*] = [1,200]×[0,1]×[1,300]. Equation (8) implies *b*(*m*, *β*) = *x*^2^(*K* − *m* − 2*x*)/(*x*^2^ + *mk*), which corresponds to the Hopf bifurcations. Equation (2) represents a plane of transcritical bifurcations.  Figure 2 shows a three-parameter bifurcation diagram. The equilibrium *E*_1_ is stable/unstable in the region to the right/left of the plane of transcritical bifurcations. The equilibrium *E*^*∗*^ exists in the region to the left of the plane and is stable/unstable in the region above/under the surface of Hopf bifurcations.

From Figure 2, we conclude that the tumor is uncontrollable when *β* is extremely small, even though both tumor killing and antitumor production rates are high. This result implies that the health of the immune system activation plays a role in fighting the cancer. Moreover, if *β* is not extremely small, tumor regression and relapse cycles later can occur through enhancing the tumor killing and antitumor cytokine rates. After repeating several cycles, the tumor can be controlled and remains at a smaller size. However, the tumor still cannot be cleared completely without treatment.

**Figure 3:**
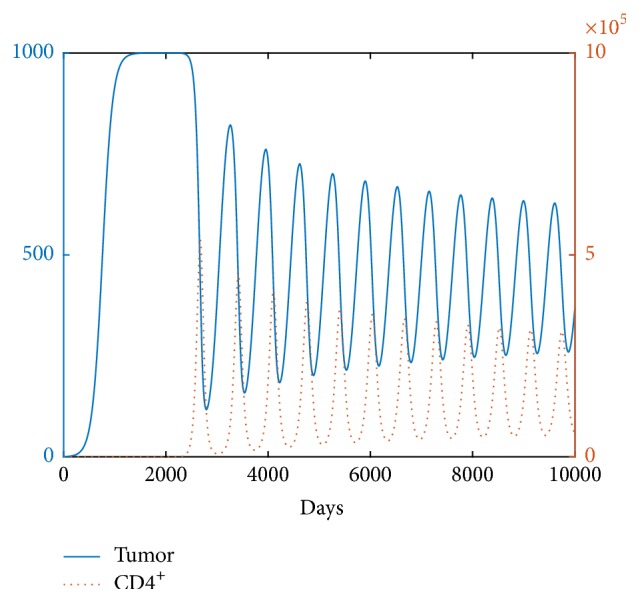
The tumor size oscillates for small *m*. Parameter values used here are the same as those in Figure 2 except *m* = 50, *β* = 0.1. The initial condition is [0.5,0.01,0].

**Figure 4:**
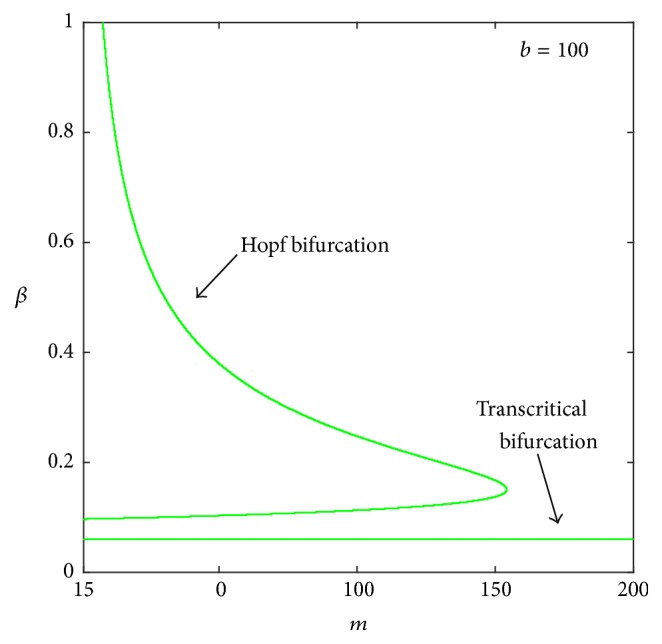
A two-parameter bifurcation diagram using *β* and *m* as bifurcation parameters at *b* = 100. Parameter values used here are the same as those in Figure 2.

We also investigate the aforementioned phenomenon from different viewpoints. The tumor oscillation for a low tumor killing rate *m* can be clearly seen from Figure 3. Moreover, we confirm our results by using *β* and *m* as two bifurcation parameters in Figure 4. For *b* = 100, a two-parameter bifurcation diagram shows that a limit cycle exists for *β*_0_ < *β* < *β*_1_. The interval of existence of the limit cycle decreases with an increase in *m*. A large tumor mass occurs when *β* is extremely small (*β* < *β*_*c*_). Notably, the numerical bifurcation curve in Figure 4 validates the theoretical result of (9).

**Figure 5:**
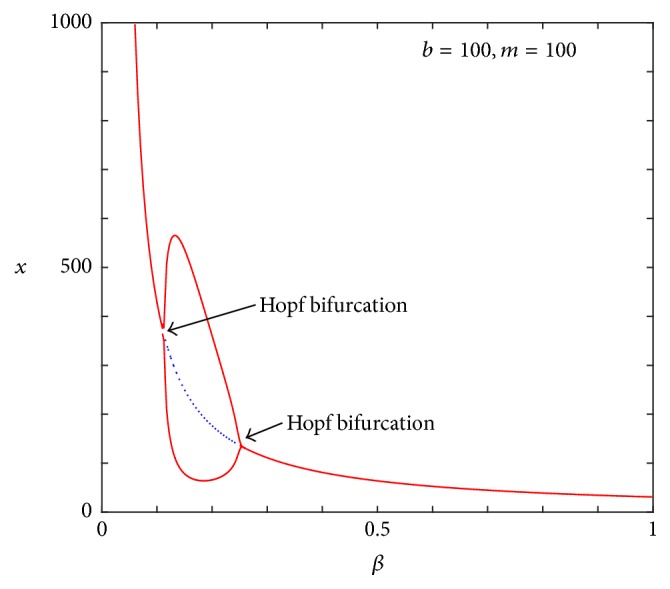
A phase parameter bifurcation diagram using *β* as the bifurcation parameter. Parameter values adopted here are the same as those in Figure 2.

A one-bifurcation parameter by using *β* as the bifurcation parameter is also shown in Figure 5. When *b* = 100 and *m* = 100, the size of the tumor decreases overall as the strength of *β* increases. Moreover, the system undergoes a Hopf bifurcation at *β* = 0.113 and 0.247. The amplitude of the limit cycle decreases and then disappears. However, the tumor cannot be eradicated without treatment. Notably, the result of this numerical one-bifurcation parameter diagram agrees with the analytical result by Anderson et al. [15] for this moderate value of *m* (*m* = 100). Figure 5 also confirms the results of the three-parameter bifurcation diagram of Figure 2.

Thus, the model does not allow for the eradication of the tumor in the nontreatment case. For this reason, we now study the role of strengthening the immune response through immunotherapy.

## 4. Immunotherapy

Because recent active immunotherapeutic approaches have used cytokine either alone or jointly with adoptive immunotherapy [25] and vaccination injections are administered repeatedly, in general [26, 27], we explore the effects of periodically pulsed therapies with either CD4^+^ T cells or Th2-related antitumor cytokine IL-4 alone or them together. Mathematically, we assume the therapy is administered at a dosage of *d* every *τ* days during a patient's lifetime. We administer either *l*_1_ or *l*_2_ or both to the tumor site, where *l*_1_ represents the implementation of CD4^+^ T cells and *l*_2_ indicates the implementation of IL-4. The discussion of injection of IL-4 to the tumor site can be seen in [28]. The external infusions *l*_1_ and *l*_2_ are modelled by Dirac Delta functions as (10) and (11), where *d*_CD4^+^_ and *d*_IL4_ are dosages of CD4^+^ T cells and IL-4 per infusion, respectively.

The parameter values are taken from [15] and listed in Table 2. These parameter values have been derived from data in the literature. The parameter for the carrying capacity *K* was derived by Kronik et al. [29]. The parameter value for the intrinsic growth rate *r* of the tumor was calculated from its doubling time by Kronik et al. [29] and Plesnicar et al. [30]. The loss rate of IL-4 *μ* was estimated from its half-life by Conlon et al. [31] and Perez-Diez et al. [12]. The values of the apoptosis *a* of CD4^+^ T cells ranging from 0.01 to 0.18 are adopted from various references [16, 29, 32]. Other parameter values are from Anderson et al. [15] with *μ* = 50, *r* = 0.027, and *a* = 0.02.

The choice of cancer treatment depends on many factors, including the stage and grade at diagnosis, the dosage and frequency of administration, and the strength of a patient's immune system. A treatment strategy may be effective for one patient but fail for another. However, the actual dosage and frequency of T cells and IL-4 are not easily determined because of the wide variation across clinical studies [29]. Because most of the treatment periods have been weekly, we set the treatment cycle to be one week and adopted the estimation of doses of CD4^+^ T cells and IL-4 from [33–35].

(10) l1t=∑n=0∞dCD4+δt−nτ,

(11) l2t=∑n=0∞dIL4δt−nτ.

In Section 2, we showed that the antigenicity of the tumor (*β*) is a key parameter. For the nontreatment case, the tumor tends to reach its maximum burden with very low values of *β* and remains at a small size for large values of *β*. The critical value is approximately *β*_*c*_ = 0.0202. Therefore, we will present two realizations representing low (*β* = 0.015 < *β*_*c*_) and high tumor antigenicity (*β* = 0.1 > *β*_*c*_) in our numerical studies. Furthermore, we will theoretically explore the stability of the tumor-free solution for each case because it indicates whether a treatment can prevent tumor recurrence after attempting to remove the tumor. We will first study the stability of the tumor-free fixed point and its dynamics when CD4^+^ T cells are administered alone.

### 4.1. Adoptive Cellular Immunotherapy Alone (*l*_1_ > 0, *l*_2_ = 0)

Define the map *F*(*X*_0_, *Y*_0_, *Z*_0_) = (*X*(*τ*), *Y*(*τ*), *Z*(*τ*)) to be the solution of (1) and (10) at *t* = *τ* with initial condition (*X*_0_, *Y*_0_, *Z*_0_).

**Theorem 2 .**

Let *l*_1_(*t*) = ∑_*n*=0_^*∞*^*d*_CD4^+^_*δ*(*t* − *nτ*). Then, the tumor-free fixed point (0, *y*_0_, 0) is unstable, where *y*_0_ = *d*_CD4^+^_/(1 − *e*^−*aτ*^).

**Proof**

Consider the tumor-free case where *x*(*t*) = 0. From (1) and (10), we have *z*(*t*) = 0, and *y*(*t*) satisfies

(12) y′t=−ayt,ynτ=ynτ−+dCD4+,n=0,1,2,….

The tumor-free fixed point (0, *y*_0_, 0), where *y*_0_ = *d*_CD4^+^_/(*e*^*aτ*^ − 1), satisfies *y*_0_ = *y*(*nτ*^−^) for *n* = 0,1, 2,…. The periodic solution $\tilde{y}(t)$ satisfying (12) with $\tilde{y}(0)=y_{0}+d_{CD4^{+}}$ is given by $\tilde{y}(t)=\left(d_{CD4^{+}}/\left(1-e^{-a\tau }\right)\right)e^{-a(t-n\tau )}$ for *t* ∈ [*nτ*, *n*(*τ* + 1)), *n* = 0,1, 2,…. To study the stability of the tumor-free fixed point (0, *y*_0_, 0), we assume *x* = *ϵT*(*t*) + *O*(*ϵ*^2^), $y=\tilde{y}(t)+\epsilon C(t)+O(\epsilon^{2})$, and *z* = *ϵE*(*t*) + *O*(*ϵ*^2^). So, *x*′(*t*) = *ϵT*′(*t*) + *O*(*ϵ*^2^). From (1), we have *T*′(*t*) = *rT*(*t*). A small tumor grows with time at a rate *r*. Therefore, the tumor-free fixed point (0, *y*_0_, 0) is unstable.

**Figure 6:**
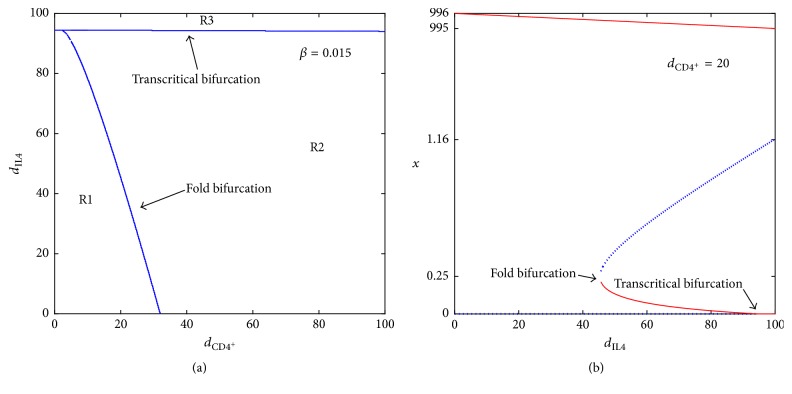
(a) The bifurcation diagram for *β* = 0.015. (b) The various dynamics at *d*_CD4^+^_ = 20.

The numerical investigations for *l*_1_ > 0 and *l*_2_ = 0 with *β* = 0.015 and *β* = 0.1 are shown in Figures 6(a) and 10(a), respectively. From Figure 6(a), the case of low tumor antigenicity, it shows that if a small dosage of CD4^+^ T cells is delivered alone, the tumor will grow to a larger size (*E*_3_^*∗*^). The model exhibits bistability with a large dosage of CD4^+^ T cells. The tumor can be either controlled to a small size or grown close to its maximum mass. This result indicates that the long-term fate of the tumor with the treatment of CD4^+^ T cells depends on the size when it is detected. For high antigenicity of the tumor, a globally closed curve exists when the dosage of CD4^+^ T cells is small. The amplitude of the closed curve decreases with an increase in the dosage of CD4^+^ T cells. As the dosage increases further, the oscillations spiral to a unique globally stable fixed point *E*_1_^*∗*^, leading to a small, persistent tumor, as shown in Figure 10(a). In general, the immunotherapy does not allow for the complete clearance of the tumor when CD4^+^ T cells are administered alone.

We now explore the tumor-free stability condition and the dynamics of periodically pulsed therapy when IL-4 is administered independently.

### 4.2. Immunotherapy with IL-4 Alone (*l*_1_ = 0, *l*_2_ > 0)

The following theorem demonstrates the tumor-free stability condition when *l*_1_ = 0, *l*_2_ > 0.

**Theorem 3 .**

Let *l*_2_(*t*) = ∑_*n*=0_^*∞*^*d*_IL4_*δ*(*t* − *nτ*). Then, the tumor-free fixed point (0,0, *z*_0_) is stable if *d*_IL4_ > *rτμm*/*δ*, where *z*_0_ = *d*_IL4_/(1 − *e*^−*μτ*^).

**Proof**

Consider the tumor-free case where *x*(*t*) = 0. From (1) and (11), we have *y*(*t*) = 0, and *z*(*t*) satisfies

(13) z′t=−μzt,znτ=znτ−+dIL4,n=0,1,2,….

Similar to the proof in Theorem 2, we have *z*_0_ = *d*_IL4_/(1 − *e*^−*μτ*^) and $\tilde{z}(t)=\left(d_{IL4}/\left(1-e^{-\mu \tau }\right)\right)e^{-\mu (t-n\tau )}$ for *t* ∈ [*nτ*, *n*(*τ* + 1)), *n* = 0,1, 2,…. To study the stability of the tumor-free fixed point (0,0, *z*_0_), we assume *x* = *ϵT*(*t*) + *O*(*ϵ*^2^), *y* = *ϵC*(*t*) + *O*(*ϵ*^2^), and $z=\tilde{z}(t)+\epsilon E(t)+O(\epsilon^{2})$. From (1), we have $T^{\prime }(t)=rT(t)-\left(\delta /m\right)T(t)\tilde{z}(t)$. Solving for *T*(*t*), the solution at *t* = *τ* is given by *T*(*τ*) = *T*(0)*e*^*rτ*−*δd*_IL4_/*mμ*^. The stability condition satisfies *T*(*τ*) < *T*(0). Therefore, the tumor-free fixed point (0,0, *z*_0_) is stable if *d*_IL4_ > *rτμm*/*δ*.

From a study by Anderson et al. [15], we know that the tumor-free state is stable if *l*_2_ > *rμm*/*δ* for the continuous injection of *l*_2_. For the periodically pulsed therapy, the stability condition is *l*_2_ > *rKτμm*/*α*. Using the parameter values in Table 2, the threshold for the stable tumor-free fixed point is *l*_2_ > 94.5 cm^3^. This dosage is seven times larger than that used in the continuous infusion of IL-4 [15]. Figures 6(a) and 10(a) also show that the tumor can be suppressed completely if the dosage is above 94.5 cm^3^. When the dosage is less than 94.5 cm^3^, the tumor will grow to a size near its carrying capacity for a patient with low tumor antigenicity or oscillate for a patient with high tumor antigenicity.

In the next subsection, we study whether the combined therapy provides a more effective treatment for curing a malignant tumor.

### 4.3. Immunotherapy with Both CD4^+^ T Cells and IL-4 (*l*_1_ > 0, *l*_2_ > 0)

Consider joint periodically pulsed therapy, where a dose of CD4^+^ T cells at *d*_CD4^+^_ and a dose of IL-4 at *d*_IL4_ are given. One cycle of the treatment is Δ*t* = *τ* days. The proof of the stability for the tumor-free fixed point is similar to those of Theorems 2 and 3 and will be omitted.

**Theorem 4 .**

Let *l*_1_(*t*) = ∑_*n*=0_^*∞*^*d*_CD4^+^_*δ*(*t* − *nτ*) and *l*_2_(*t*) = ∑_*n*=0_^*∞*^*d*_IL4_*δ*(*t* − *nτ*). Then, the tumor-free fixed point (0, *y*_0_, *z*_0_) is stable if *d*_IL4_ > *rτμm*/*δ*, where *y*_0_ = *d*_CD4^+^_/(1 − *e*^−*aτ*^) and *z*_0_ = *d*_IL4_/(1 − *e*^−*μτ*^).

We will now explore how the combined pulsed treatment of CD4^+^ T cells and IL-4 affects the system numerically. In general, for both *β* = 0.015 and *β* = 0.1, there are three different regions and four steady states, at the most. We denote these regions as *E*_0_, *E*_1_^*∗*^, *E*_2_^*∗*^, and *E*_3_^*∗*^, respectively, where *E*_0_ is the tumor-free steady state and *E*_*i*_^*∗*^ is a positive steady state, where *i* = 1,2, 3. The tumor size increases with the subindex for the positive steady state. Note that when the treatment of adopting CD4^+^ T cells is used, the amount of CTL ranges from 1.06 cm^3^ to 72.61 cm^3^ per infusion, according to [15]. Therefore, we explore the dynamics of the combined pulsed treatment for *β* = 0.015 and *β* = 0.1, with dosages ranging from 0 to 100 cm^3^. The discussion for a low antigenicity (*β* = 0.015) case follows.

#### 4.3.1. Dynamics and Biological Implications for *β* = 0.015

There are mainly three categories for the dynamics from Figure 6(a). As the amount of CD4^+^ T cells increases from R1 to R2, the fold bifurcation occurs. By contrast, the system undergoes transcritical bifurcation when the amount of IL-4 augments from part of R1/R2 to R3. Here is a list showing the dynamics and biological implications for each region.

**Figure 7:**
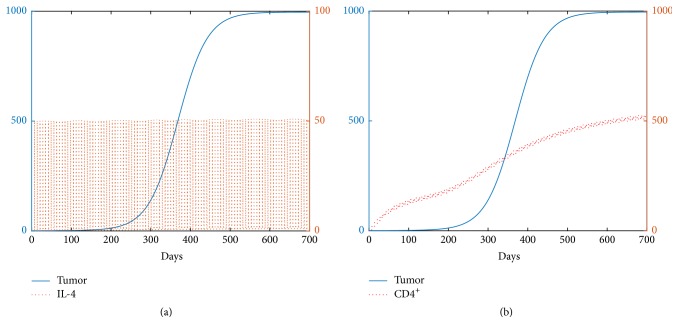
The pictures (a) and (b) represent the evolution of the tumor for *β* = 0.015. The initial condition is [0.5,0.01,0].

**Figure 8:**
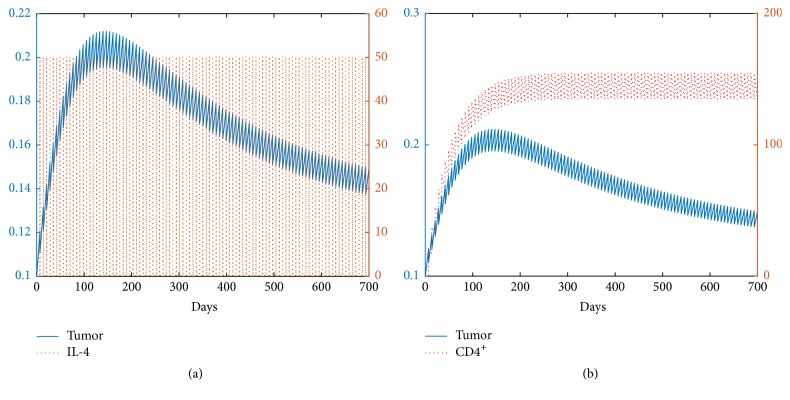
The pictures (a) and (b) represent the evolution of the tumor for *β* = 0.015. The initial condition is [0.1,0.01,0].

- Two steady states, *E*_0_ and *E*_3_^*∗*^, exist in region R1. In R1, the range of CD4^+^ T cells is from 0 to 32 cm^3^ and the range of IL-4 is from 0 to 94.5 cm^3^ per injection. *E*_3_^*∗*^ is the only stable fixed point and the tumor growth is uncontrollable.
- As the amount of CD4^+^ T cells increases from region R1 to R2, two additional fixed points, *E*_1_^*∗*^ and *E*_2_^*∗*^, are born due to the fold bifurcation; see Figure 6(a), where *E*_1_^*∗*^ is stable. In R2, two stable fixed points *E*_1_^*∗*^ and *E*_3_^*∗*^ exist. Tumor burden can either be controlled to a very small size (0.0422 cm^3^) (Figure 8) or grow to near its carrying capacity (991.3218 cm^3^) (Figure 7). However, the tumor cannot be eradicated completely.  Figure 6(b) shows changes in dynamics for *d*_CD4^+^_ = 20 as the level of IL-4 changes. At *d*_CD4^+^_ = 20, the fold bifurcation occurs as the level of IL-4 increases from R1 to R2 and the transcritical bifurcation occurs as the amount of IL-4 changes from R2 to R3.
- R3 is a region where the dosage of IL-4 is very large. Three steady states, *E*_0_, *E*_2_^*∗*^, and *E*_3_^*∗*^, exist, where *E*_0_ and *E*_3_^*∗*^ are stable. Therefore, the solution will tend to either *E*_0_ (the tumor-free state) or *E*_3_^*∗*^ (the tumor survives; the tumor size is close to its maximum burden) depending on the initial condition. This result means that the tumor can be cleared only if the tumor size is small when it is detected. Consequently, if the tumor can be detected earlier, the immunotherapy may succeed in clearing the tumor.

#### 4.3.2. Dynamics and Biological Implications for *β* = 0.1

**Figure 9:**
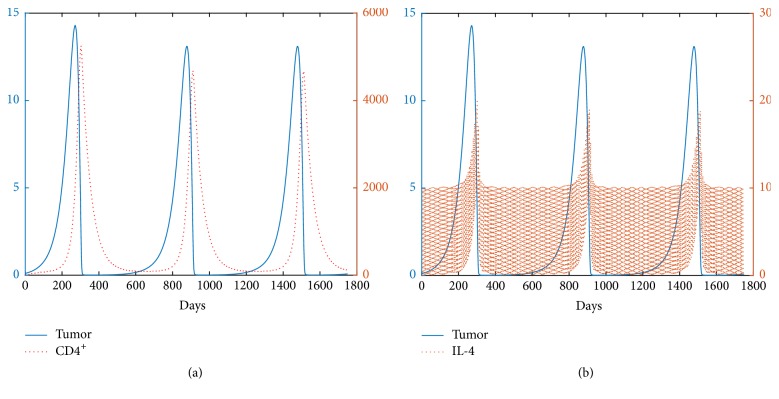
The pictures (a) and (b) represent the time evolution of the tumor for *β* = 0.1. The initial condition is [0.1,0.01,0].

**Figure 10:**
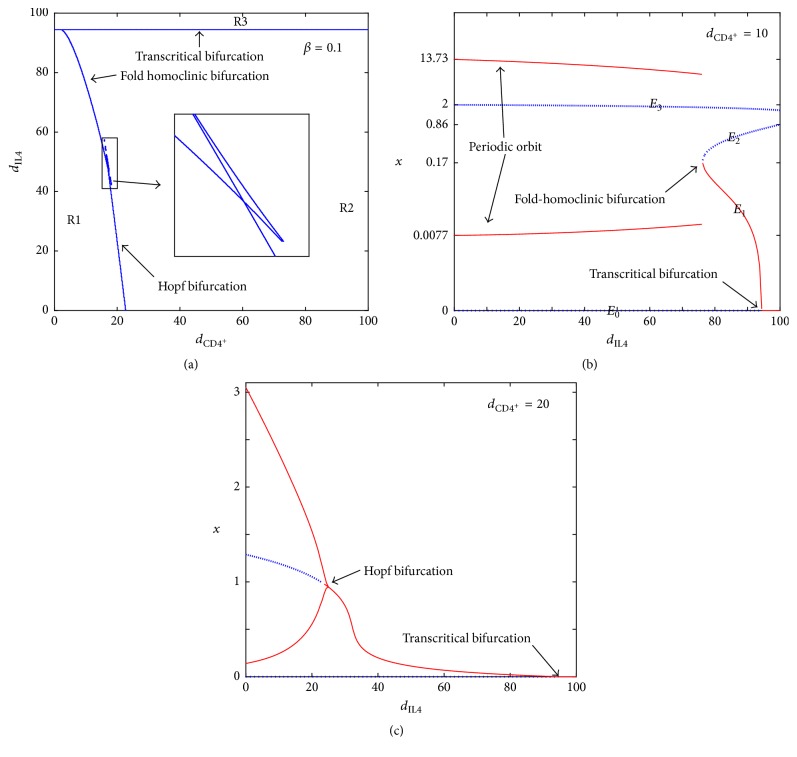
(a) The bifurcation diagram for *β* = 0.1. (b) The detailed development of the fold-homoclinic bifurcation. (c) The change of dynamics at *d*_CD4^+^_ = 20.

**Figure 11:**
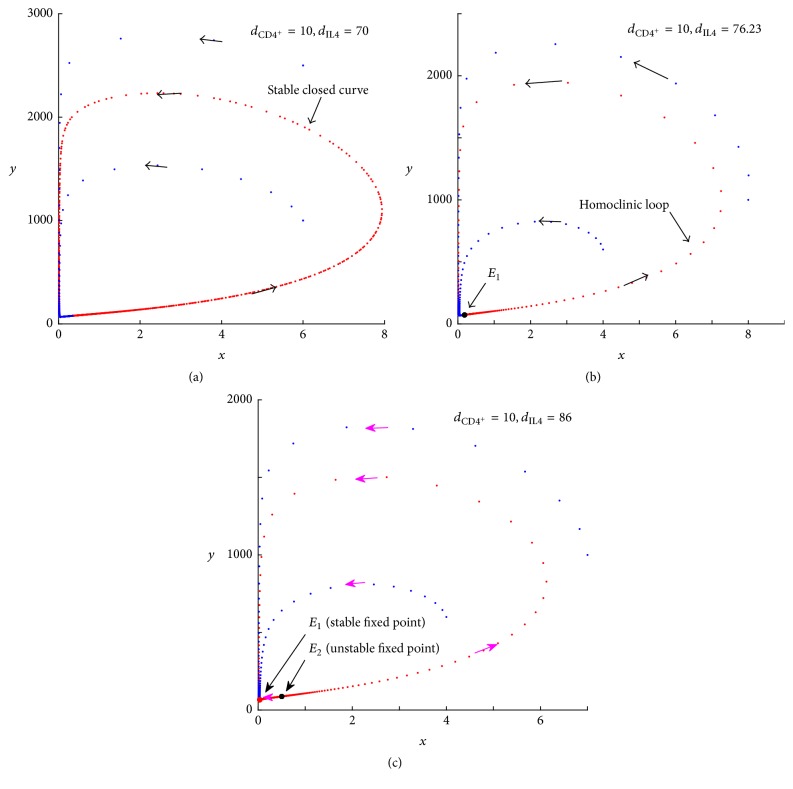
The evolution of the fold-homoclinic bifurcation for *β* = 0.1, CD4^+^ T = 10. (a) A stable closed curve exists when IL-4 < 70. (b) A nonhyperbolic fixed point (*E*_1_^*∗*^) appears on the curve IL-4 = 76.23, and the curve becomes homoclinic to *E*_1_^*∗*^. (c) *E*_1_^*∗*^ splits into a saddle (*E*_2_^*∗*^) and a stable node (*E*_1_^*∗*^) when IL-4 > 76.23. All orbits are attracted to the curve under the map *F*.

For a patient with high tumor antigenicity *β*, polytherapy is more effective. The bifurcation diagram in Figure 10(a) shows the different dynamics involved with various dosages of CD4^+^ T cells and IL-4. As the amount of CD4^+^ T cells increases from the lower/upper part of R1 to R2, the Hopf/fold-homoclinic bifurcation occurs. By contrast, the system undergoes a transcritical bifurcation when the dosage of IL-4 augments from R1 or R2 to R3. The dynamics and the biological relevance are presented as follows:

- *E*_0_ is unstable and *E*_1_^*∗*^ is a stable closed curve in the region R1. The tumor size oscillates in this region (Figure 9). A critical value of IL-4_*c*_ exists. Below/above IL-4_*c*_, a Hopf/fold-homoclinic bifurcation occurs at the intersection of R1 and R2; see Figures 10(a) and 10(b). When the system undergoes the fold-homoclinic bifurcation, the closed curve breaks and *E*_1_^*∗*^ develops to a stable node; see Figures 10(a) and 10(b). The evolution of the fold-homoclinic bifurcation is shown in Figure 11. All orbits tend to curve under the iterations of *F* from Figure 11. This result means that, with the help of immunotherapy, the tumor will spend a portion of the cycle near its maximal burden and the rest with a mass close to zero. Eventually, the tumor is controlled at a small dormant state.  Figure 10(c) shows the profile of these diverse dynamics at *d*_CD4^+^_ = 20 as the amount of IL-4 crosses the different regions.
- In region R2, the only stable fixed point *E*_1_^*∗*^ is a stable node. Immunotherapy can help to suppress the tumor to a very small size.
- As we continue increasing the dosage of IL-4 from R1 to R3, the system experiences a transcritical bifurcation; see Figure 10(a). The stable state *E*_1_^*∗*^ in R1 is lost and the tumor-free steady state *E*_0_ becomes stable in R3. Thus, the tumor can be eradicated completely in the region of R3.

## 5. Results and Discussion

In this paper, we studied the effects of the periodically pulsed immunotherapy of CD4^+^ T cells and Th2-related tumor suppression cytokine (IL-4) on tumor-immune interactions. Without treatment, for a patient with very low tumor antigenicity *β*, the tumor will grow to its carrying capacity even when both the tumor killing *m* rate and antitumor production rate *b* are high. By contrast, tumor recurrence can be observed and its mass can be controlled to a smaller size if *m* or *b* is high and *β* is not extremely small. The tumor cannot be eradicated without any treatment.

The effects of periodically pulsed administration with either CD4^+^ T cells or the cytokine IL-4 or both are studied. The administration of the cytokine IL-4 alone can suppress the tumor completely for any level of tumor antigenicity when the dosage of cytokine is above 94.5 cm^3^. When the level of IL-4 is less than 94.5 cm^3^ and a patient has low tumor antigenicity, immunotherapy fails to stop tumor growth. However, if tumor antigenicity is high, a stable closed curve exists and a patient will experience tumor regression and relapse. Notably, these stable closed curves have not been observed in the case of low tumor antigenicity.

With the periodically pulsed treatment of CD4^+^ T cells alone, the tumor-free state is not stable and the tumor cannot be eradicated. When an average or a large number of CD4^+^ T cells are infused, the tumor can be reduced to a smaller size for a patient with high tumor antigenicity. The tumor can be either controlled to a very small size or raised near to its maximum burden for a patient with low tumor antigenicity. This result suggests that the tumor can be regulated by administering CD4^+^ T cells alone for a patient having a strong immune system or whose tumor has been diagnosed at an early stage.

Our study shows that periodically pulsed treatment with IL-4 may be more effective either as a monotherapy or along with the CD4^+^ T cell treatment. Although implementing a high dose of IL-4 can clear the tumor, this treatment may overboost the immune system and have a detrimental effect on the patient. These side effects may outweigh the benefits of tumor eradication. The treatment with CD4^+^ T cells alone does not provide a satisfactory outcome; the tumor-free state does not exist for any level of tumor antigenicity. Nevertheless, an average dose of CD4^+^ T cells is sufficient to maintain the tumor at a small dormant state. Together with cytokine IL-4, the amount of CD4^+^ T cells can be reduced to maintain the tumor at a smaller size within host. Thus, the combined effects may be the best option.

The tumor microenvironment is exceedingly complex. Many issues are worthy of discussion. For example, what is the optimal treatment strategy if we administer drugs for some periods and use various dosages? From our study, administering CD4^+^ T cells alone cannot clear the tumor. Nevertheless, research has shown that a blockade of CTL-associated antigen 4 (CTLA-4) on T cells can heighten in vivo cytotoxicity and improve the antitumor activity, including killing a well-established large tumor [36–40]. Further investigations of tumor-reactive CD4^+^ T cells with cytotoxic activities for more complicated models may provide prominent advantages for the treatment of human malignancies.

## Figures and Tables

**Table 1:** Parameters, biological meanings, and units.

Parameter	Biological meaning	Unit
*r*	Intrinsic growth rate of the tumor	day^−1^
*K*	Carrying capacity of the tumor	cm^3^
*δ*	Maximum tumor killing rate by antitumor cytokine	day^−1^
*m*	Half saturation constant of the tumor killing rate	cm^3^
*β*	Maximum CD4^+^ T cell production rate (antigenicity of the tumor)	day^−1^
*k*	Half saturation constant of CD4^+^ T cell production rate	cm^3^
*a*	Death rate of the CD4^+^ T cells	day^−1^
*α*	Maximum production rate of the antitumor cytokine	day^−1^
*b*	Half saturation constant of antitumor cytokine production rate	cm^3^
*μ*	Antitumor cytokine loss rate	day^−1^
*l*_1_(*t*)	Treatment of CD4^+^ T cells	cm^3^ day^−1^
*l*_2_(*t*)	Treatment of cytokine (IL-4)	cm^3^ day^−1^

**Table 2:** Parameter values.

*α*	*a*	*b*	*δ*	*k*	*μ*	*m*	*K*	*r*
0.1	0.03	0.1	0.1	10	50	1	1000	0.027
